# Human colorectal mucosal microbiota correlates with its host niche physiology revealed by endomicroscopy

**DOI:** 10.1038/srep21952

**Published:** 2016-02-26

**Authors:** Ai-Hua Wang, Ming Li, Chang-Qing Li, Guan-Jun Kou, Xiu-Li Zuo, Yan-Qing Li

**Affiliations:** 1 Department of Gastroenterology, Shandong University, Qilu Hospital, Jinan, 250012, China; 2 Department of Gastroenterology, Shandong Rongjun General Hospital, Jinan, 250013, China

## Abstract

The human gut microbiota plays a pivotal role in the maintenance of health, but how the microbiota interacts with the host at the colorectal mucosa is poorly understood. We proposed that confocal laser endomicroscopy (CLE) might help to untangle this relationship by providing *in vivo* physiological information of the mucosa. We used CLE to evaluate the *in vivo* physiology of human colorectal mucosa, and the mucosal microbiota was quantified using 16 s rDNA pyrosequencing. The human mucosal microbiota agglomerated to three major clusters dominated by Prevotella, Bacteroides and Lactococcus. The mucosal microbiota clusters did not significantly correlate with the disease status or biopsy sites but closely correlated with the mucosal niche physiology, which was non-invasively revealed by CLE. Inflammation tilted two subnetworks within the mucosal microbiota. Infiltration of inflammatory cells significantly correlated with multiple components in the predicted metagenome, such as the VirD2 component of the type IV secretory pathway. Our data suggest that a close correlation exists between the mucosal microbiota and the colorectal mucosal physiology, and CLE is a clinically available tool that can be used to facilitate the study of the *in vivo* correlation between colorectal mucosal physiology and the mucosal microbiota.

The human gastrointestinal tract harbors a complex microbial community (microbiota), and the comprising bacteria account for more than 90% of the cells in the human body^1^. The gut microbiota confers multiple benefits for the host including education of the immune system, nutrition utilization and niche protection^2^. The disturbance of the gut microbial community, which is often termed “dysbiosis,” has been linked to diseases such as obesity^3^, autism^4^, inflammatory bowel disease^5^,^6^, and irritable bowel syndrome^7^. Unlike our own genomes, our microbiomes are inherently dynamic^8^. Although the composition of the gut microbiota has been widely documented in recent years, we still have little insight into the microbiota dynamics. Understanding how the microbiota interacts with the physiological and pathological features in the mucosa has become a tantalizing question and a prerequisite to effectively modulate the gut microbiota.

The gut microbiota is highly affected by the host physiology. The human large intestine harbors approximately 10^14^ bacteria, and the members in such a dense community have to fiercely compete for limited energy sources from the bowel contents^9^. Changes in the host mucosa physiology, such as inflammation, would generate oxidation products that serve as additional electron acceptors, resulting in the outgrowth of facultative anaerobic bacteria^10^. Additionally, the goblet cells in the intestinal epithelium secret mucin glycans that feed the mucosa associated microbiota^9^, and the density and function of the goblet cells are often reduced in colitis and neoplastic lesions. The disturbances of host physiology would cause dysbiosis in the gut microbiota, which in turn might participate in pathogenesis. However, detailed studies on the correlation between the mucosal microbiota and mucosal physiology in human cohorts are still lacking.

A confocal laser endomicroscope (CLE) is a clinically available tool that could provide both *in vivo* histological images and insights into epithelial physiology. Multiple physiological aspects of the intestinal mucosa, such as the epithelial integrity^11^,^12^, vascularization^13^,^14^, and inflammatory activity of ulcerative colitis^15^ could be evaluated using CLE. Recent studies documented the usefulness of CLE to evaluate mucosal responses to a food antigen^16^. A major advantage of CLE is that the *in vivo* evaluation is *in situ* and non-invasive; thus, the microbiota of the targeted mucosa could be sequenced and quantified. Therefore, in this study, we utilize the advantage of the probe-based CLE in combination with 16S rDNA pyrosequencing to evaluate the host physiology and mucosal microbiota, respectively. We aimed to analyze 1) how the mucosal microbiota was disturbed under pathological conditions, 2) whether the dysbiosis was associated with specified host mucosal physiological alternations.

## Methods

### Subjects and sample collection

Patients requiring colonoscopy in Qilu Hospital (Jinan, China) were recruited for this study from November 2013 to April 2014. The inclusion criteria required that subjects be between 18 and 80 years old, and both inpatients and outpatients were included. The exclusion criteria include the following: antibiotic usage within 2 months, probiotic or prebiotic (such as inulin) usage within 2 months, ascites, jaundice, liver cirrhosis, impaired renal function, coagulopathy, fever, pregnancy, breast feeding, inability to provide informed consent, and a known allergy to fluorescein sodium. All of the participants provided written informed consent. The protocol was approved by the Institutional Ethics Committee of Qilu hospital and registered at ClinicalTrials.gov with Identifier NCT02063919. The registion date was January 29, 2014. The procedures were conducted in accordance with the approved guidelines.

### CLE imaging and mucosa feature grading

Preparation before pCLE was the same as conventional colonoscopy. Endoscopic procedures were performed by one of the three endoscopists (G.-J.K., C.-Q.L. and X.-L.Z.) who were experienced in pCLE (>100 pCLEs). The colon was examined using the EPK-i high-definition white-light colonoscope (Pentax, Tokyo, Japan). Before pCLE examination, 6 ml of 10% fluorescein sodium was intravenously injected, and within the following 2–5 minutes, the lesions were examined using the confocal laser probe (Cellvizio, Mauna Kea Technologies, Paris, France). If multiple lesions were found in one patient, only the most distal lesion was characterized using CLE because 1) the primary goal was to study the correlation between the mucosal microbiota and host niche physiology, 2) better image stability could be obtained in distal lesions. The CLE videos were viewed by an endoscopist (A.-H.W.) in a real-time manner and reviewed within 1 day to ensure the accuracy of the results. For the lesions enrolled in this study, the diagnosis was made based on the combination of white light and CLE images according to published criteria^15^,^17^,^18^. A control site near the lesion was evaluated using CLE and was biopsied in the same way. The exact CLE evaluated site was biopsied, washed in aseptic normal saline 3 times, snap frozen in liquid nitrogen, and stored at −80 °C. The clinical disposition of the patients was based on the conventional histology results from other biopsy samples.

**Figure 1:**
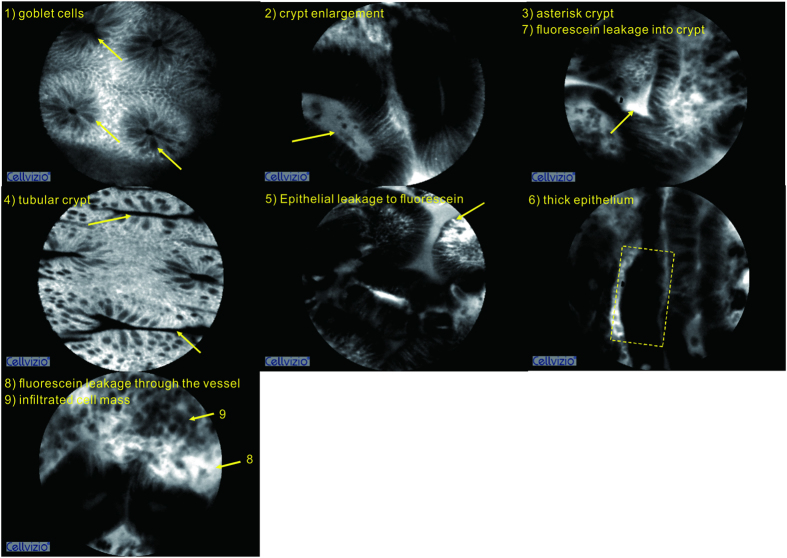
The environmental factors of the host mucosa visualized by endomicroscopy. An example of each factor is indicated by arrows or box.

The host mucosa niche physiology was evaluated using CLE. Nine environmental factors of the host mucosa were graded as revealed by CLE: 1) density of goblet cells, 2) crypt enlargement, 3) asterisk crypt, 4) tubular crypt, 5) epithelial leakage to fluorescein, 6) thick epithelium, 7) fluorescein leakage into crypt, 8) fluorescein leakage through the vessel, and 9) infiltrated cell mass. The density of the goblet cells was graded at three levels (0, few or no; 1, reduced; 2, normal), and all of the other features were graded at two levels (0 for absence and 1 for presence) in a non-redundancy analysis (RDA). The example of each factor on the CLE images was shown in Fig. 1.

### DNA extraction and sequencing

The biopsied mucosal samples were transferred to Majorbio (Shanghai, China) where the total DNA was extracted, amplified and sequenced according to their standardized protocol^19^. The V3- >V1 region of the 16S ribosomal subunit gene was amplified using 27F/533R barcoded primers and sequenced using a Roche Genome Sequencer FLX+). The sequencing results were archived in the Short Reads Archive (PRJNA285379).

### Raw sequencing data processing, diversity, cluster analysis, and PCoA analysis

Raw sequencing data were prepared using Mothur v 1.33.0^20^ according to their proposed 454 SOP (http://www.mothur.org/wiki/454_SOP). The raw sff files were decoded, denoised, trimmed and then aligned to Silva references (Release 119) using the default parameters. The sequences were clustered with the same operational taxonomy unit (OTU) if their distances were less than 0.03. Each OTU was assigned a taxonomy using the classify.otu command and further represented by the finest taxonomy name. The OTU table was converted to biom files, and the taxa relative abundances at domain to genus levels were generated using the summarize_taxa.py command in QIIME v1.8.0.

### Diversity, cluster analysis, and PCoA analysis

The Shannon index and Chao index were calculated to indicate the microbiota diversity in each sample. Each index was compared between groups using the Kruskal-Wallis test (KW) in SAS V.9.3. The cluster analysis based on the Euclidean distance was conducted based on the relative abundances of all OTUs without scaling in R 3.1.1. The correlation between the cluster and disease status, or between two samples clustering within one patient, was tested using chi-square test in SAS. A primary coordination analysis was performed based on the Braycurtis distance using dist.shared and pcoa command sequentially in Mothur. The pcoa coordination with respect to the diagnosis and cluster result was plotted in R. The pcoa coordination was re-plotted with samples from the same connected patient.

### LEfSe analysis and non-redundancy analysis

LEfSe (linear discriminant analysis [LDA] coupled with effect size measurements) analysis was conducted to calculate the biomarkers between the groups. A stricter one-to-one comparison was adopted to filter biomarkers. To explore the correlation between mucosa niche features and the mucosal microbiota, RDA was performed using the calibrate package in R. The graded mucosa features were input as environmental factors and plotted along with samples and taxonomy.

### Network analysis

The co-occurrence network of the top 40 abundant OTUs, and the environmental factors evaluated by CLE, was visualized to explore the host-microbiota interaction. The Spearman correlation between the OTU abundances was calculated using a different cor.test function in R. For the relationship between the OTUs, a strict p-value threshold (*P* ≤ 0.001 and r > 0.6) was applied to filter the strong correlations. A less strict threshold (p ≤ 0.05) was adopted to filter out the significant correlations between the environmental factors and OTUs. The combined result was exported to Cytoscape V.3.2.1. Each node represents an OTU involved in the microbiota network, whereas the solid and dashed edge represents positive and negative correlations, respectively. The nodes were positioned according to the weighted perfuse force directed layout.

### Metagenome prediction and correlation to environmental factors

The metagenome of the colorectal microbiota was predicted using PICRUSt^21^. Briefly, the above 16 S sequences were re-agglomerated to OTUs with a similarity cutoff value of 99% and further blasted against GreenGenes (version 13_5). The OTU table was exported in biom format using Mothur. Then, the OTU table was normalized by dividing each OTU by the known/predicted 16S copy number abundance. The normalized OTU abundance values were then multiplied by the respective predicted gene counts for the metagenome prediction. The predicted functional pathways were annotated using the Clusters of Orthologous Groups (COG) database. Then, a Spearman correlation of the predicted COG and the environmental factors were calculated in R, and significant correlations (p < 0.005) were exported to Cytoscape for visualization.

## Results

### Patient demographics and clinical features

A total of 69 diseased and control mucosal sites in 39 patients were finally analyzed in this study. The demographics of these sites are summarized in Table 1, and the details of the UC patients are listed in Table S1. Most of these mucosal sites were sampled from sigmoid colon (22/69) and rectum (30/69). CLE could clearly visualize the intestinal mucosa, and key local physiology factors were graded based on the *in vivo* images (Fig. 1). A total of 25 of the mucosal sites were diagnosed as adenoma, and 5 were diagnosed as ulcerative colitis. A total of 30 pairs of mucosa biopsies were sampled from these diseased sites and the nearby control sites, and an additional 9 samples were biopsied from the patients diagnosed as normal under CLE.

### Diversity of mucosal microbiota

**Figure 2:**
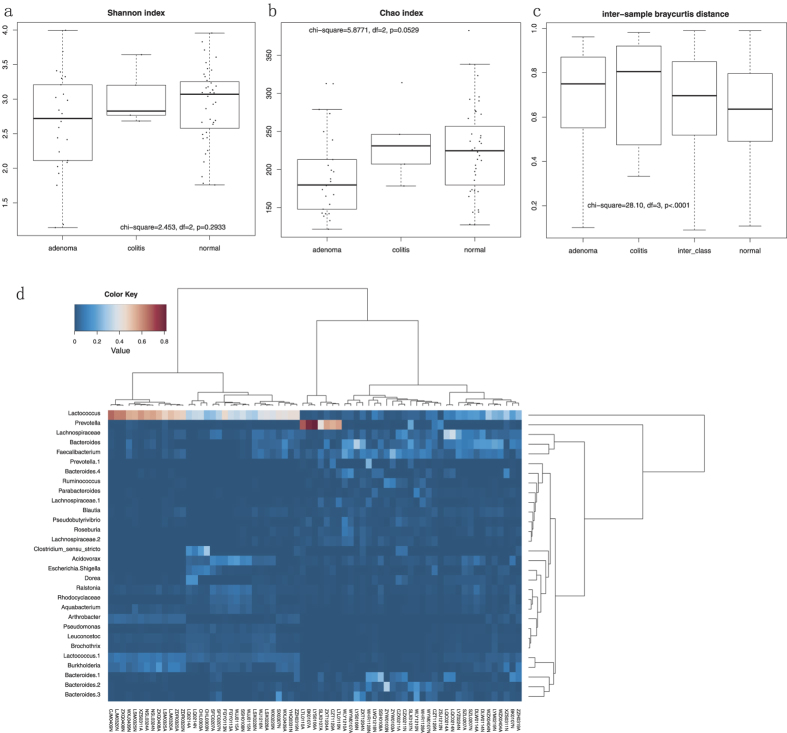
The sample diversity and heatmap plot. Shannon index (**a**) and Chao (**b**) index were compared among the disease statuses. The pairwise Bray-Curtis distance (**c**) was calculated for the distances within each group and among groups. (**d**) The heatmap plot of the 25 most abundant OTUs in all of the sequenced samples. The OTUs were named by their best fit taxa.

A total of 554,070 reads were finally analyzed after sequence de-noising, trimming and chimera picking. The sequencing data was deposited in the Short Reads Archive under project PRJNA285379. These reads were clustered into 1,418 OTUs. We first checked the sequencing depth by plotting the rarefaction curve for each sample (Fig. S1). Most of the samples reached their plateaus, which suggested the adequacy of the sequencing. We compared the mucosal microbial diversity between the disease statuses using a Shannon and Chao index. We found no differences in the Shannon index among adenoma, ulcerative colitis, and normal mucosa samples (Fig. 2a). The microbiota on the adenoma mucosa tended to have a lower Chao index than that on the ulcerative colitis and control mucosa (KW, χ^2^ = 5.8771, df = 2, p = 0.0529, Fig. 2b).

We then asked whether the microbiota on the diseased mucosa were heterogeneous due to the potential loss of the host control. We calculated the Bray-Curtis distance between samples and compared these distances within and between each group. We found that the difference was significant (KW, χ^2^ = 28.10, df = 3, p < 0.0001, Fig. 2c). The intra-adenoma distance and the inter-class distance were significantly higher than the intra-normal distances (p < 0.05 for both). We plotted the 25 most abundant OTUs in a heat-map plot, and we found that Lactococcus and Prevotella are the most abundant OTUs in most samples (Fig. 2d).

### Major clusters of colonic mucosal microbiota

**Figure 3:**
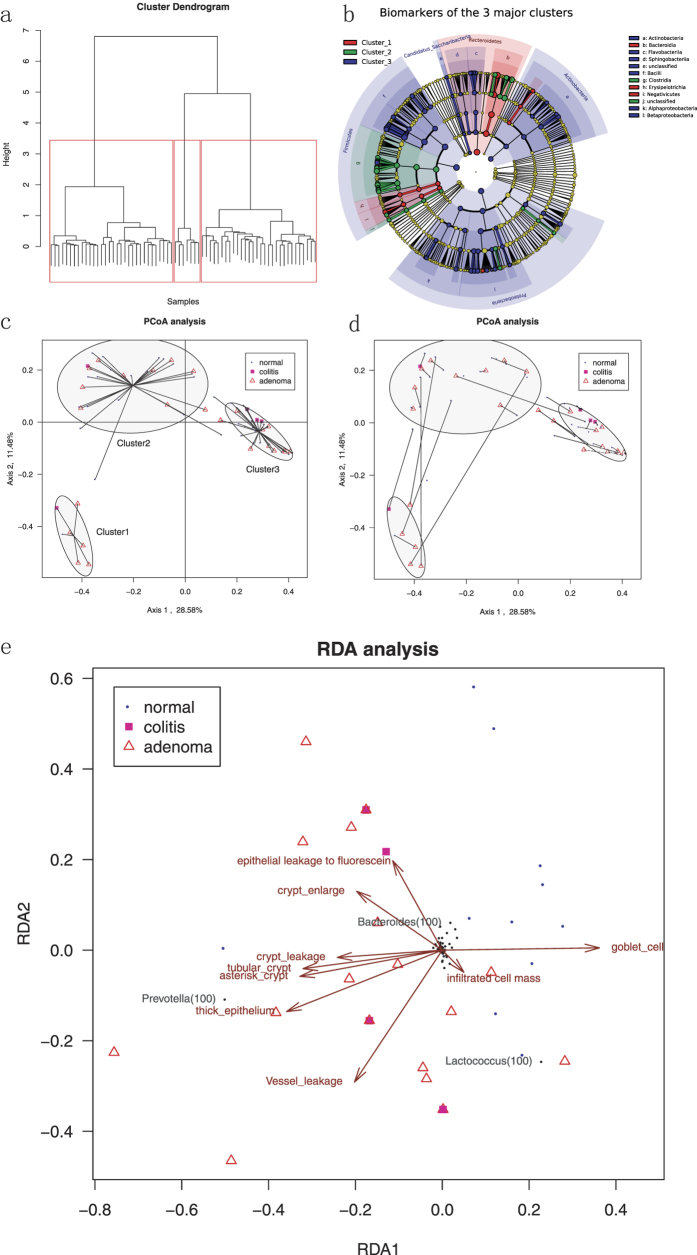
The major clusters of colorectal mucosal microbiota. (**a**) The cluster analysis revealed 3 deep-rooted clusters of mucosal microbiota. See also Fig. S2. (**b**) The biomarkers for the three major clusters were highlighted in the cladogram. From the innermost to outmost, each cycle represents the phylum, class, order, family, and genus level. Each dot represents a taxon, and its diameter positively correlates with the abundance of the corresponding taxon. The colored and shadowed dots indicate the biomarkers for each cluster. See also Fig. S2. (**c**) The PCoA plot of the mucosal microbiota with samples from the same connected microbiota cluster. The ellipse was estimated to cover 75% of the dots in this group. (**d**) The PCoA plot of the mucosal microbiota with samples from the same connected patients. (**e**) The RDA plot of the samples, OTUs and host mucosal physiology. Black dots indicate OTUs named by their corresponding genus name. Brown arrows indicate host mucosal physiology factors. See also Fig. S3.

We checked whether the mucosal microbiota were clustered by performing a cluster analysis. When all of the samples in the study were clustered based on the abundance of all of the variable taxa, three deep-rooted clusters were found (Fig. 3a).

We next evaluated the features in the three major mucosal microbiota clusters. We first analyzed the biomarkers for the three clusters using the LEfSe method. A total of 118 taxa were included to distinguish the three microbiota clusters (Fig. 3b, Table S2). We found that Cluster 1 was associated with significantly higher Bacteroidetes. Prevotella was the most prominent genus level biomarker for Cluster 1 (LDA score = 5.518,p = 2.58*10^−5^), and thus, Cluster 1 might be designated the Prevotella dominant cluster. The other biomarkers for cluster 1 included Megamonas, Pseudobutyrivibrio, Butyricimonas, and Collinsella. For Cluster 2, Bacteroides were the most prominent genus level biomarker (LDA score = 5.056, p = 2.07*10^−10^). Thus, cluster 2 might be designated as the Bacteroides dominant cluster. Other biomarkers for cluster 2 included Parasutterella, Haemophilus, and members in Lachnospiraceae and Ruminococcaceae. For Cluster 3, Firmicutes was the most abundant phyla, and Lactococcus was the most prominent genus level biomarker (LDA score = 5.374, p = 4.64*10^−12^). Thus, Cluster 3 was designated Lactococcus dominant. When plotting the phylum level abundances, a clear-cut demarcation was found between the clusters (Fig. S2). This indicated that the human mucosal microbiota was not evenly distributed but formed 3 discontinued clusters dominated by Prevotella, Bacteroides and Lactococcus.

### Correlation of mucosal microbiota to disease statues

We next investigated whether the major clusters of the mucosal microbiota were associated with diseases. We calculated the PCoA coordinates and plotted them with respect to the disease status and major clusters (Fig. 3c). The PCoA confirmed the agglomeration of the mucosal microbiota into the 3 major clusters. The correlation between the disease statues and the clusters were insignificant (χ^2^ = 6.496, df = 4, p = 0.165, Table 2). No significant correlation existed between the mucosa biopsy locations and the microbiota clusters (χ^2^ = 13.10, df = 8, p = 0.1084).

To investigate whether the microbiota from the diseased and control mucosa of the same patient tend to fall in the same cluster, we re-plotted the PCoA coordinates with the samples from the same connected patients (Fig. 3d). We found that the microbiota on the 30 disease-control paired sites tended to fall in the same cluster (χ^2^ = 22.73, df = 4, p < 0.0001). To investigate whether any microbiota cluster tended to be more deviant from its paired lesions, we calculated the correlation between the microbiota cluster consistency within the same patients and the control or the diseased site cluster. We found no correlation between the microbiota cluster consistency and the control site cluster (χ^2^ = 2.825, df = 2, p = 0.2436). However, the mucosal microbiota on the diseased site tended to be different from the corresponding control sites if the diseased site occurred in cluster 1 (χ^2^ = 12.65, df = 2, p = 0.0018, Table 2, Fig. 3d).

### Mucosal niche physiology correlated with the mucosal microbiota

We next investigated whether the mucosal microbiota interacts with the host niche physiology. Here, we used the RDA analysis to visualize the relationship among the disease statues, OTUs and host niche physiological factors (Fig. 3e). The niche factors were plotted with brown arrows, and each factor’s weight was proportional to its arrow length. The normal mucosa tended to have a higher goblet cell density, whereas the adenoma mucosa was associated with an asterisk or tubular crypt, thick epithelium and leaky vessels. This was consistent with our previous findings^22^. From the RDA figure we see that the Prevotella, Bacteroides and Lactococcus were the OTUs that most correlated with the niche factors. The Bacteroides negatively correlated with the inflammatory cell (Spearman r = −0.3365, p = 0.00470) and the crypt leakage (Spearman r = −0.2888, p = 0.0161). These results suggest that the mucosal microbiota correlated with its host niche physiology.

We also plotted the RDA coordination of all of the samples with the samples grouped by disease statues (Fig. S3). We found no clear-cut boundaries to separate the disease statues. This was consistent with Table 2, which together indicated the lack of overall microbial demarcation with separate microbiota on healthy, adenoma and inflammatory mucosa.

### Interaction network between the epithelial environment and the microbiota

**Figure 4:**
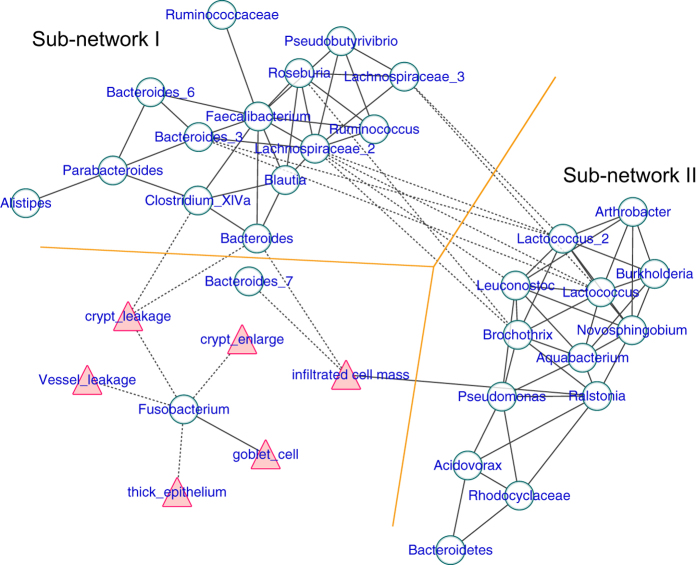
The co-occurrence network of the colorectal mucosal microbiota and the local epithelial environmental factors. Each green round node represents an OTU, and each red triangle represents an environment factor revealed by CLE. The solid and dashed edge represents a positive and negative correlation, respectively. The whole network could be divided into two subnetworks, where the positive correlation exists within each subnetwork, and the trans-subnetwork correlation was negative.

Because we found correlations between the mucosal microbiota and the epithelial niche factors, it was rational to further evaluate how the mucosal microbial community interacts with the local environment at the colorectal epithelium. Thus, we performed a network analysis. We calculated the correlation between the abundance of the pairwise OTUs and screened the strong correlations with p < 0.001 and r > 0.6. The correlation between the niche factors and OTUs was filtered by a loose threshold of p < 0.05 because of the graded input of these factors. The overall result was displayed in Fig. 4, and the detailed list is presented in Table S3. Clear visualization showed that the mucosal microbiota formed two major mutualistic subnetworks (Subnetwork I and Subnetwork II in Fig. 4). The correlations were all positive within each subnetwork, and the correlations between different subnetworks were all negative. The Ruminococcus, Faecalibacterium, Bacteroides and unclassified Lachnospiraceae were the central OTUs within Subnetwork I. The Lactococcus, Aquabacterium, Brochothrix and Leuconostoc were the central OTUs within Subnetwork II.

Significant co-variance existed between the mucosal microbiota and the corresponding local epithelial physiology. The infiltrated cell mass correlates negatively with the Bacteroides in Subnetwork I and positively with Ralstonia in Subnetwork II (Fig. 4, Table S3). Fluorescein leakage into the crypt negatively correlated with the Bacteroides and Clostridium XIVa in subnetwork I. Fusobacterium strongly correlated with multiple environmental factors, but it had little interaction with the rest of the microbiota (Fig. 4, Table S2).

### Correlation between predicted metagenome environmental factors

**Figure 5:**
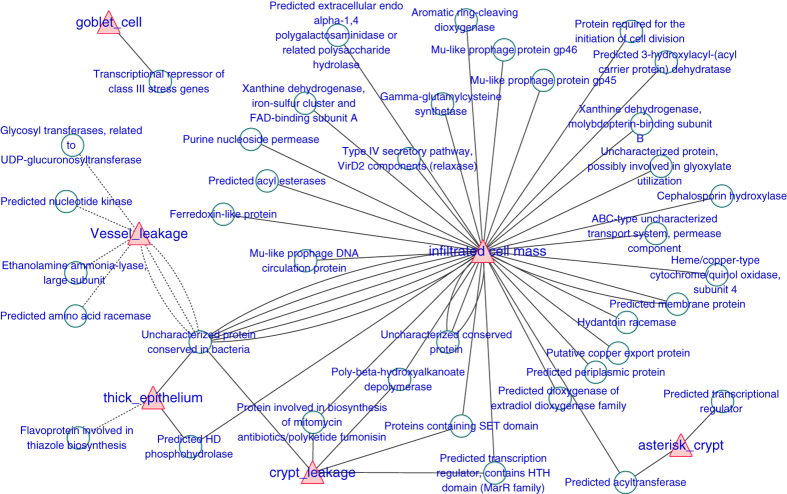
Interactions between the predicted metagenome and the local epithelial environmental factors. Each red triangle represents an environment factor revealed by CLE. Each green round node represents a COG pathway. The solid and dashed edge represents positive and negative correlations, respectively.

Finally, we examined whether the gene repertoire of the mucosal microbiota interacts with the epithelial local environments. We predicted the metagenome and analyzed the COG using PICRUSt^21^. Significant correlations (p < 0.005) were visualized in Fig. 5 and the detailed list is presented in Table S4. We found that the inflammatory cell infiltration most strongly interacts with the metagenome components such as the Type IV secretory pathway, Mu-like prophage, xanthine dehydrogenase, polygalactosaminidase, purine nucleoside permease, and hydantoin racemase (Fig. 5, Table S4). This network indicates that extensive interaction exists between the local epithelial environment and the predicated metagenome.

## Discussion

In this study, we used endomicroscopy to evaluate the *in vivo* physiology of the host colorectal mucosa and its correlations to the mucosal microbiota. We discovered the following: 1) Endomicroscopy could clearly visualize the *in vivo* pathophysiology of the colorectal mucosa, which closely interacted with the mucosal microbiota. 2) Members of the mucosal microbiota formed two subnetworks based on the co-occurrence relationship, and the inflammatory activity oppositely correlates with the two subnetworks. 3) The interaction between the mucosal microbiota may involve multiple orthologous groups of the metagenome, which provides new targets for further mechanistic study.

To our knowledge, this is the first study exploring the correlation between the human colorectal mucosal microbiota and the *in vivo* host mucosal physiology. This was difficult for the conventional *in vitro* or *ex vivo* physiology evaluation approaches (e.g., an ussing chamber) because we could hardly evaluate the *in vivo* physiology while sequencing the microbiota at the same site. CLE was invented in 2005 and can provide images of subcellular resolution^23^. It has been widely used as a tool for *in vivo* diagnosis and screening of early neoplasia. CLE can visualize and distinguish the epithelial gaps and goblet cells in the human intestine^24^. CLE can also visualize the mucosal physiology *in vivo*, which may serve as an indication for disease severity or the risk of relapse^25^,^26^. Patients with UC and Crohn’s disease had a significantly increased density of gaps, and the leakage of the fluorescein into the lumen through the epithelial gaps could be directly visualized under pCLE^24^,^27^. Recent studies also used CLE to evaluate the food-associated changes in the intestinal mucosa of patients with irritable bowel syndrome^16^. We also tracked the MSC homing to the colonic mucosa using *in vivo* endomicroscopy in rat model sets^28^. In this study, we non-invasively studied the mucosal physiology using CLE. We conclude that CLE is a useful tool to facilitate the study of the *in vivo* interactions between the colorectal mucosal physiologies.

The human microbiota has already been extensively studied using fecal samples. Previous studies have found that the human fecal microbiota agglomerated to three robust discontinuous clusters dominated by Prevotella, Bacteroides, and Ruminococcus, respectively^29^. Compared with the fecal microbiota, the mucosal microbiota was closely attached to the host mucosa and less disturbed by the fluctuations of the luminal environment^30^. Whether human colorectal mucosal microbiota would likely cluster to several robust clusters had not been previously investigated. In our study, the mucosal microbiota formed three major clusters dominated by Bacteroides, Prevotella and Lactococcus. However, we consider this information insufficient to draw a general conclusion that the whole human colorectal mucosal microbiota was similarly clustered. Despite the rapid accumulating data on the fecal microbiota by projects such as American Gut Project^31^, data on the mucosal microbiota is growing slowly. Additional research meta-analyzing all of the currently available next generation sequencing data of the human colorectal mucosal microbiota is still needed.

In this study, we highlighted the interactions of the colorectal mucosal physiology with the mucosal microbiota. Our results indicated that the bacteria on the colorectal mucosa is not only a self-governing community but also affected by the host physiology. The alternations of the host mucosa, such as the epithelial leakage to fluorescein or the infiltrated cell mass, may directly affect the growth rate of certain strains and indirectly affect the interaction networks between the strains. A very recent study found that oxygen and nutrients provided by the host tissue affect the composition of the intestinal microbiota^32^. Our study described the result of dynamic shifts *in vivo* under different disease conditions. In the network analysis, we found that the mucosal microbiota could be divided into two subnetworks based on the co-occurrence relationship. Furthermore, the inflammatory results favor Subnetwork II and negatively correlated with Subnetwork I through Ralstonia and Bacteroides, respectively. However, the cross-sectional data in this study limited the determination of the directional interactions. Additional studies of the longitudinal changes of the mucosal microbiota and simulation of the microbial dynamics are warranted.

Understanding the mucosal microbial dynamics would provide useful insight for microbiota targeted treatment. The *Fusobacterium* was invasive to gut epithelial cells and was already documented to be involved in the pathogenesis of colorectal adenoma^33^,^34^ and inflammatory bowel disease^35^,^36^. In this study, we found that *Fusobacterium* was less involved in the rest of the mucosal microbiota, but it was more closely correlated with the local epithelial environment. In contrast, Clostridium *difficile*, a common pathogen in antibiotic induced dysbiosis, was documented to closely interact with multiple members of the microbiota such as Blautia^37^. The invasion of *C. difficile* requires a significantly disturbed and susceptible microbiota, which is a so-called “niche opportunity”^37^. The isolated role of Fusobacterium that was revealed in our network analysis may partially explain that Fusobacterium may prosper and invade the host from the roughly normal microbiota, which was the case in acute appendicitis^38^,^39^,^40^. A detailed mechanism of how Fusobacterium colonize the diseased epithelium merits further study.

This study highlighted that an extensive correlation exists between the mucosal metagenome and the local epithelial environment, especially inflammatory cell infiltration. The VirD2 components of the type IV secretory pathway significantly and positively correlated with infiltrated cell mass that was visualized by CLE. The bacterial type IV secretory pathway translocates DNA and protein substrates to bacterial or eukaryotic target cells generally by a mechanism dependent on direct cell-to-cell contact^41^. They are a strong antigen recognizable by dimeric immunoglobulin A^42^. Many Gram-negative bacterial pathogens deliver potentially hundreds of virulence proteins to eukaryotic cells for modulation of different physiological processes during infection^41^. Previous studies found that establishment of systemic Brucella *melitensis* infection through the digestive tract requires the type IV secretion system^43^. However, little is known about the role of the type IV secretory pathway in colorectal inflammation. Whether the increased type IV secretion system in the mucosal microbiota triggered inflammation and its detailed mechanisms requires further study.

In this study, samples were biopsied in a paired manner from adenoma and UC patients. The apparently normal site near the diseased lesion served as an internal paired control. In the PCoA and cluster plot (Fig. 3d), the diseased site and the corresponding control site might fall into different clusters despite the spatial closeness. Normal cases (n = 9) were also included in the study cohort to provide an external control. Their enrollment could exclude the effect of potential systemic disease factors on the mucosal microbiota, morphology, and physiology.

In this study, the morphologic and physiologic data of the colorectal mucosa was obtained through subjectively assessing the *in vivo* pCLE images. Although these assessing methods had been reported by previous studies, they might be less precise than the real quantitative test such as the Ussing chamber test. However, regarding this explorative study, evaluating pCLE images has two advantages: 1) pCLE evaluation is completely non-invasive and does not require any biopsy specimens. 2) Multiple indexes could be simultaneously evaluated at the same targeted site that was later biopsied for microbial profiling. Otherwise, several different lesions around the target must be biopsied for different examinations. Thus, we deem this approach as an acceptable compromise between clinical feasibility and accuracy. We also emphasize that the correlations warrant further mechanistic study, and until then, the mucosal morphology or physiology should be evaluated by specific and real quantitative experiments.

Several other limitations merit discussion in this study. First, only the 16s rDNA amplicon was sequenced and the metagenome of the mucosal microbiota was inferred using PICRUSt. Further shotgun sequencing studies exploring the mucosal microbiota function under different conditions are needed. Then, the adaptive immune function of the host mucosa was an important factor shaping the landscape of the microbiota, but it was not directly evaluated in this study. A more comprehensive study evaluating the host IgA concentration and the host transcriptome (in addition to the microbiota) is warranted. Furthermore, the number of UC patients is small (n = 5), and we could not systematically and comprehensively profile the mucosal microbiota of UC patients.

In conclusion, CLE is a useful tool to investigate the *in vivo* correlation between the host mucosal physiology and the colorectal mucosal microbiota. A close co-variance exists between the mucosal physiology and the mucosal metagenome.

## Additional Information

**How to cite this article**: Wang, A.-H. *et al.* Human colorectal mucosal microbiota correlates with its host niche physiology revealed by endomicroscopy. *Sci. Rep.* **6**, 21952; doi: 10.1038/srep21952 (2016).

## Supplementary Material

Supplementary Information

Supplementary Table S1

Supplementary Table S2

Supplementary Table S3

## Figures and Tables

**Table 1:** Patient demographics and clinical features of the analyzed lesions.

Total number of samples/patients	69/39
Gender (male/female), n/n	20/19
Median age, years (range)	59 (31–77)
Indications of colonoscopy	
Previous history of polyp	5
Abdominal pain	9
Diarrhea without pain	1
Bleeding	6
Emaciation	2
Recurrent bowel obstruction	1
Tenesmus	1
Bloating	1
Constipation	1
Screening	12
White light plus endomicroscopic diagnosis	
Colorectal adenoma	25
Ulcerative colitis	5
Endoscopically normal	39
Biopsy Location	
Ascending colon	3
Transverse colon	4
Decending colon	12
Sigmoid colon	22
Rectum	28
Number of paired biopsies	30
Number of unpaired lesions	9

**Table 2:** The major mucosal microbiota clusters’ correlation with disease status, location, and per patient analysis.

Major Clusters in allsamples (N = 69)	CLE diagnosis			Biopsy location				
	Adenoma	Colitis	Normal	Ascending	Transverse	Descending	Sigmoid	Rectum
Cluster 1	5	1	1	2	0	0	2	3
Cluster 2	10	1	19	0	2	5	10	13
Cluster 3	10	3	19	1	2	7	10	12
Statistics	Chi-square = 6.496, df = 4, p = 0.165			Chi-square = 13.10, df = 8, p = 0.1084				
Major Cluster’s samplesfrom the same patient(N = 30)	Control site cluster			Diseased site cluster				
	Cluster 1	Cluster 2	Cluster 3	Cluster 1		Cluster 2	Cluster 3	
In different clusters	0	6	2	5		2	1	
In the same clusters	1	9	12	1		9	12	
Statistics	Chi-square = 2.825, df = 2, p = 0.2436			Chi-square = 12.65, df = 2, p = 0.0018				
