# Supplementary material for: Human colorectal mucosal microbiota correlates with its host niche physiology revealed by endomicroscopy
Source: Sci Rep. 2016 Feb 26;6:21952. doi: 10.1038/srep21952 (PMC4768150; doi:10.1038/srep21952)
Supplement: Supplementary Information [file srep21952-s1.doc]

**Human colorectal mucosal microbiota correlates its host niche physiology revealed by endomicroscopy**

**Running head: Microbiota interacts with host physiology**

**Ai-Hua Wang**^1, 2^**, Ming Li**^1^**, Chang-Qing Li**^1^**, Guan-Jun Kou**^1^**, Xiu-Li Zuo**^1^**, Yan-Qing Li**^1*^

Affiliations:

^1^ Department of Gastroenterology, Shandong University, Qilu Hospital, Jinan, China, 250012

^2^ Department of Gastroenterology, Shandong Rongjun General Hospital, Jinan, China, 250013

Ming Li and Ai-Hua Wang contributed equally to this manuscript.

***Corresponding Author:**

**Yan-Qing Li**

Department of Gastroenterology, Shandong University, Qilu Hospital

107 Wenhuaxi Road, Jinan, China

250012

Fax: +86-531-82166090

Email: liyanqing@sdu.edu.cn

**Supplemental Figures**

**
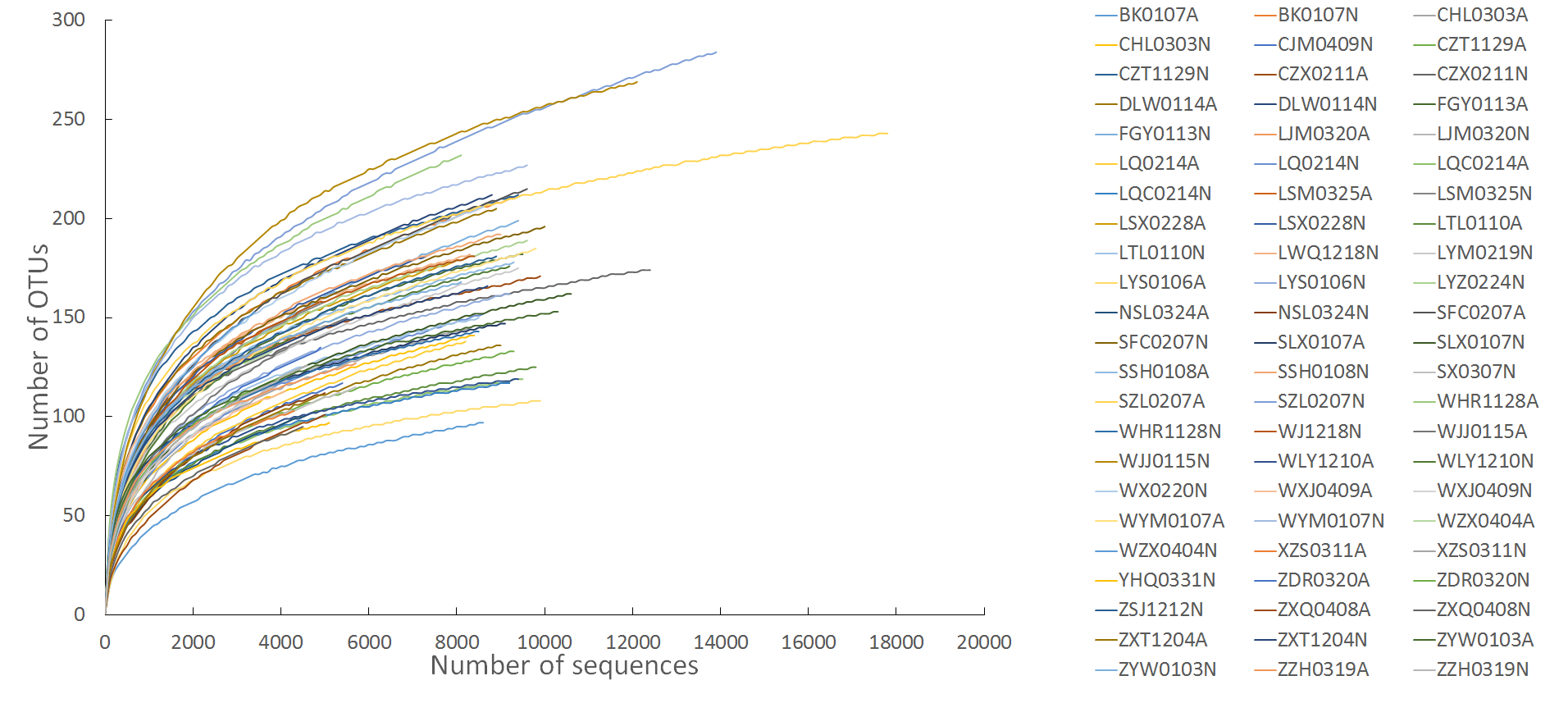
**

Figure S1. The rarefaction curve for each sample. For each sample, the number of observed OUT (Y-axis) were plotted against its number of sequences (X-axis). For each curve, reaching plateau indicates the adequacy of sequencing for this sample.


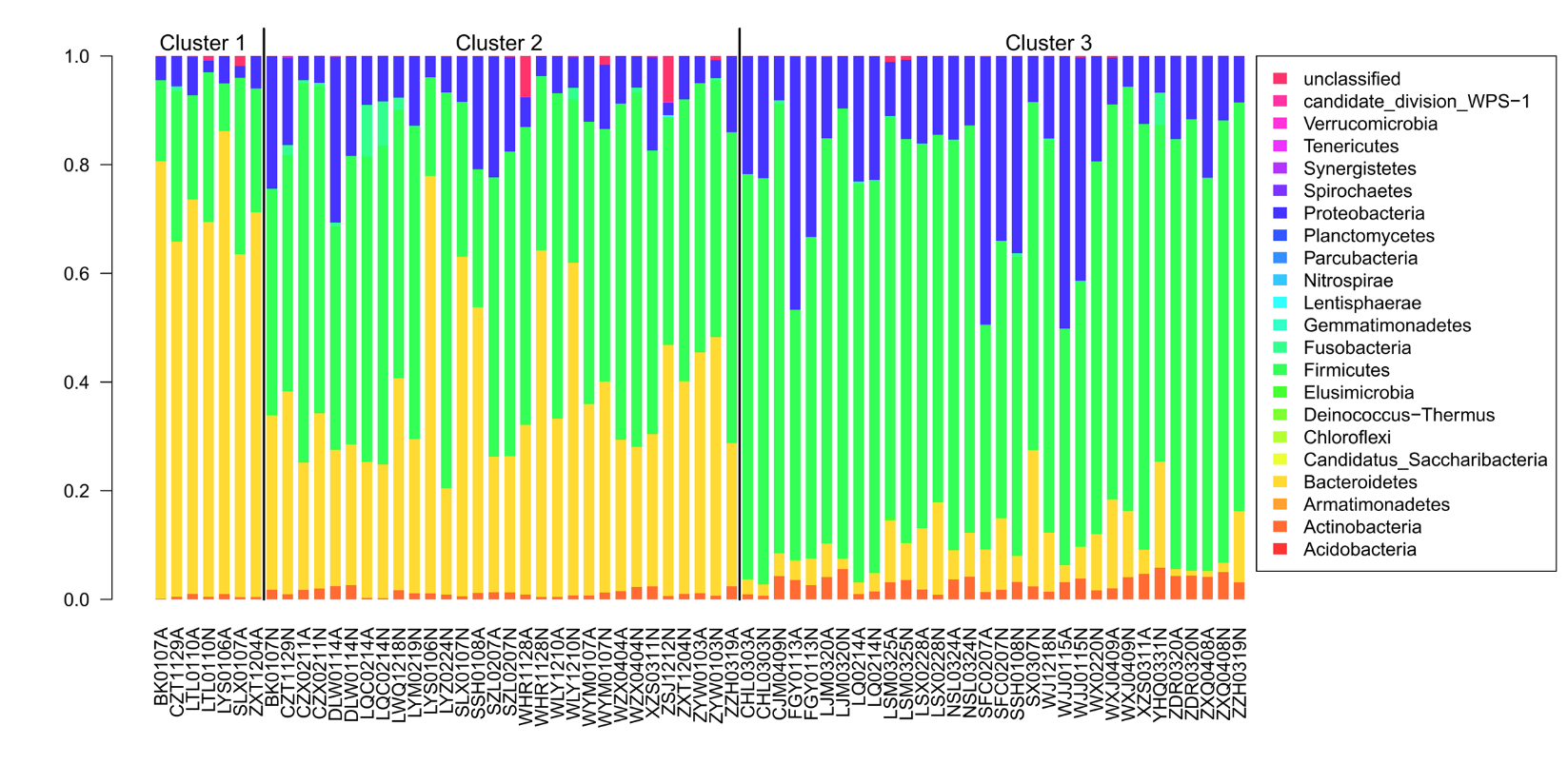


Figure S2. Bar plot of phylum abundances of colorectal mucosal microbiota. Three clear demarcated clusters was separate by vertical lines.


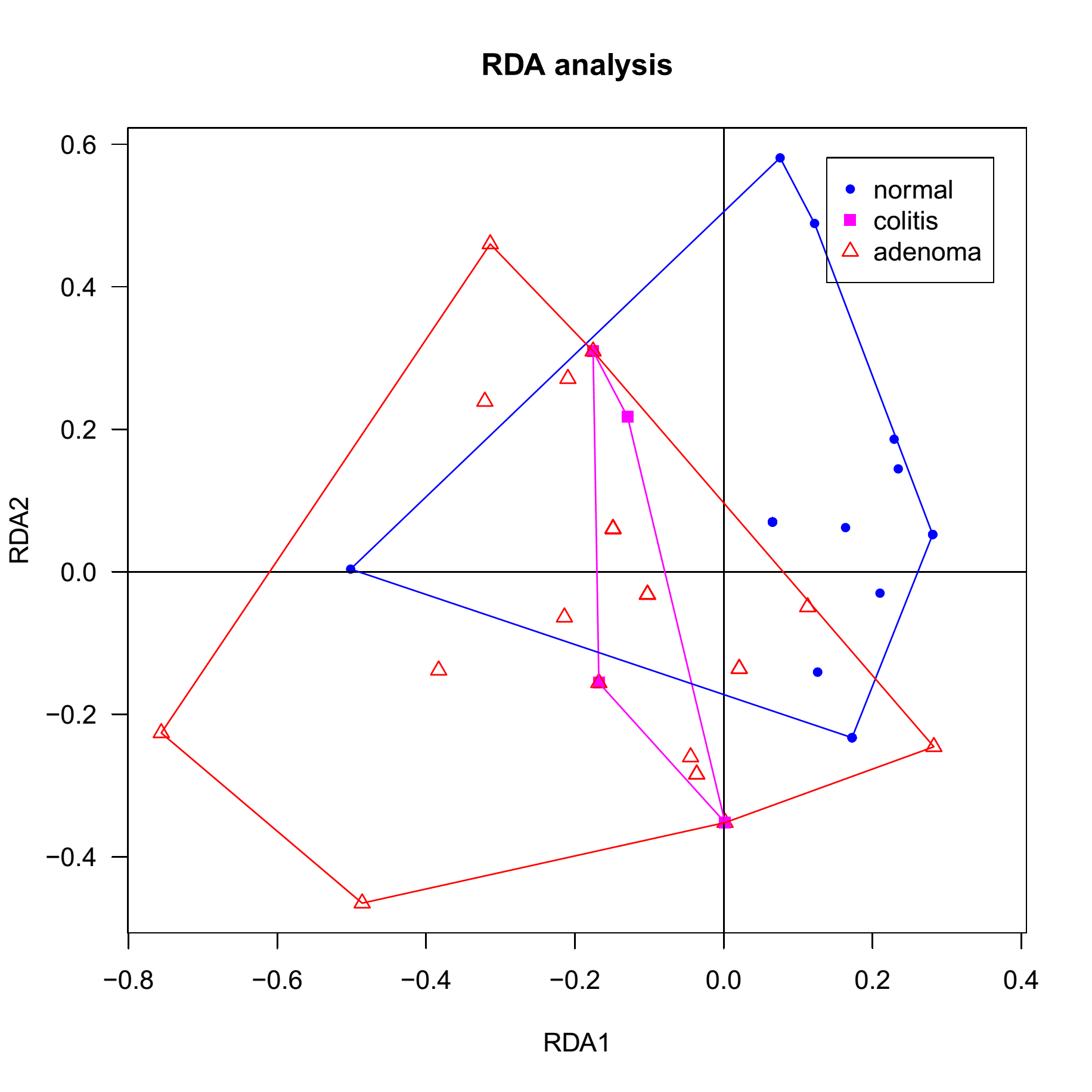


Figure S3. RDA plot with samples grouped by disease statues. No clear-cut boundaries was found to separate the disease statues.

Supplemental Tables

Table S1. Details of enrolled ulcerative colitis patients.

| Patient Code | age | sex | Diagnosis | Previous treatment | Time to last treatment |
| --- | --- | --- | --- | --- | --- |
| SLX0107 | 67 | Female | UC (rectum, active phase) | Sulfonamides, immunosuppressant | 2 month |
| WJJ0115 | 41 | Male | UC (rectum, active phase) | mesalamine | 2 week |
| SFC0207 | 39 | Female | UC (rectum, active phase) | None | NA |
| LQ0214 | 40 | Female | UC (left colon, active phase) | None | NA |
| CZX0211 | 47 | Female | UC (sigmoid to rectum, active phase) | None | NA |

UC, ulcerative colitis.

Table S2. Biomarker for 3 major mucosal clusters. The larger the linear discriminant analysis (LDA) score one taxon got, the more robust biomarker was the taxon for the respective cluster.

Table S3. The co-occurrence relationship of the mucosal microbiota OTUs and the local environment factors. The first and second column represent the OTU names and environment factors involved in the network shown in the Figure 5. The third column and fourth column represents the Spearman r and the p value respectively.

Table S4. The interactions between the predicted metagenome and the local epithelial environment factors. The first and second column represent the Clusters of Orthologous Groups (COG) pathway and environment factors involved in the network shown in the Figure 6. The third column and fourth column represents the Spearman r and the p value respectively.
